# Psychosocial well-being among individuals with chronic kidney disease undergoing hemodialysis treatment and their caregivers: a protocol of a mixed method study in Sri Lanka and Poland

**DOI:** 10.3389/fpsyg.2023.1194991

**Published:** 2023-12-07

**Authors:** Darshika Thejani Bulathwatta, Judyta Borchet, Agata Rudnik, Mariola Bidzan

**Affiliations:** ^1^Department of Psychology and Counseling, Faculty of Health Sciences, The Open University of Sri Lanka, Colombo, Sri Lanka; ^2^Institute of Psychology, Faculty of Social Sciences, University of Gdańsk, Gdansk, Poland; ^3^Academic Center for Psychological Support, University of Gdansk, Gdansk, Poland; ^4^Ateneum-University in Gdansk, Gdansk, Poland; ^5^Institute of Pedagogy and Languages, University of Applied Sciences in Elbląg, Elbląg, Poland

**Keywords:** chronic kidney disease, psychosocial well-being, shame and guilt-proneness, emotional suppression, acceptance of illness, caregivers, mixed-method study

## Abstract

Chronic Kidney Disease (CKD) can be identified as one of the non-communicable diseases (NCDs) which affect millions of people worldwide, including in Sri Lanka and Poland. The prevalence of CKD has been rising over the last three decades due to the identification of CKD with unknown etiology and the increment of NCDs such as hypertension and diabetes mellitus among the Sri Lankan population. Poland can be identified as a European country that has 4 million patients with CKD, which is the second most common chronic disease in the country. CKD is associated with the physical, economic, psychological, and social burden on patients and their caregivers. The current study is aimed to investigate the psychosocial well-being of CKD patients and their caregivers in Sri Lanka and Poland. The current study is a mixed-method study aimed to investigate the psychosocial well-being of individuals with chronic kidney disease undergoing hemodialysis and their caregivers in Sri Lanka and Poland. Participants in the quantitative part of the project will be individuals with chronic kidney disease undergoing hemodialysis from Sri Lanka (*n* = 63) and Poland (*n* = 63) who are currently undergoing hemodialysis treatment. Kidney Disease Quality of Life-Sort Form, Beck Depression Scale, Test of Self-Conscious Affect, The Courtauld Emotional Control Scale, Acceptance of Illness Scale, and a demographic and medical information sheet will be used in both Sri Lankan and Polish samples. Apart from that, the qualitative phase of the study will involve semi-structured interviews with individuals diagnosed with CKD, selected randomly from the initial part of the research, and their close relatives. Notably, the participant count will remain undetermined, as this approach addresses the inherent challenges of exploratory research.

## 1 Introduction and the background to the study

Chronic Kidney Disease (CKD) can be identified as one of the non-communicable diseases (NCDs) which affect millions of people worldwide, including in Sri Lanka and Poland, every year (Lameire et al., 2021; Adoli et al., 2022). The prevalence of CKD has been rising over the last three decades, especially among Sri Lankan farming communities, due to the identifying CKD with unknown etiology (CKDu) and the increment of NCDs such as hypertension and diabetes mellitus (Rajapakse et al., 2016; Kumaresan and Senawirathne, 2017; Kafle et al., 2019). Although the definition and the classification have evolved over time, according to international guidelines, CKD is a condition that can decrease kidney function as measured by glomerular filtration rate (GFR) of less than 60Ml/min per 1.73m^2^, or markers of kidney damage, or both and this condition needs to last for at least 3 months regardless of the underlying cause (Webster et al., 2017). Furthermore, hypertension and diabetes are the leading causes of CKD and their incidence is increasing at an alarming rate (Hernandez and Nasri, 2013; Kovesdy, 2021).

CKD is a progressive disease that does not have any warning signs. Sometimes people lose up to 90% of their kidney function before getting any symptoms, and they progress to a state that can be called end-stage kidney disease (ESKD)/end-stage renal disease (ESRD), or end-stage kidney failure (Vithange et al., 2021). According to the proposed criteria of the National Kidney Foundation (2002), CKD is divided into five stages based on the function of the individual’s kidneys, and conservative treatment is recommended until the disease reaches its fourth stage (Andrade and Sesso, 2012). However, as a consequence of the disease reaching the fifth stage (ESRD), people need artificial filtering or a kidney transplant. There are two types of artificial filtering which are called peritoneal dialysis (PD) and hemodialysis (HD). However, HD can be identified as the most frequent treatment modality in the world (Gerogianni and Babatsikou, 2014a, b; Odette Dorcas et al., 2018). HD is a treatment method in which the patient is connected to a machine that takes over the function of the damaged kidneys. This treatment method is inconvenient as the procedure is time-consuming and needs to be performed frequently - often lasts several hours and must be performed at least three times a week. Moreover, patients need to restrict their fluid intake (Lindsay et al., 2014). Hemodialysis patients’ lives are challenging not only due to the long-term nature of their diseases but also because of the nature of the treatment they are undergoing. According to a study on hemodialysis patients, the quality of life of hemodialysis patients was poor and even though hemodialysis increases patients’ lifespan, it has serious physical, psychological, and social implications that impact their quality of life and their families (de Assis et al., 2018). Hence, ESRD can be identified as a multi-dynamic health hazard as it affects significantly their family, education, financial and professional status, physical and social functioning, and mental health (Gerogianni and Babatsikou, 2014a, b).

CKD is increasing worldwide at an annual growth rate of 8% (Senanayake et al., 2017; Senanayake, 2018) and is ranked among the top 20 causes of death according to the Global Burden of Diseases (Adejumo et al., 2019). Furthermore, complications of CKD include anemia, bone disease, and increased risk of cardiovascular disease and cancer (Webster et al., 2017). Moreover, due to economically non-viable treatment methods, there is expected to be an increase of 70% in ESRD among patients in developing countries (Gunathilaka et al., 2014). Abraham et al. (2015) stated the effect of ethnicity on CKD development. According to them, the prevalence of CKD is lower in whites than in Asians. Furthermore, they have identified South Asia as a “hotspot” for developing ESRD due to a lack of managing patient registries, poor access to health care, and limited opportunities for early detection and management of the disease. Another study also highlights the high incidence of ESRD among Indo-Asian residents in the United Kingdom. This high incidence of ESRD is in part due to the prevalence of diabetic mellitus, small kidneys, unremarkable urinary sediment, and unknown etiology (Ball, 2001). Similarly, Chandie Shaw et al. (2002) found that Indo-Asian people who are residents in the Netherlands had a 40-fold risk of having ESRD due to type II diabetics compared to the native Dutch population. Moreover, according to the results of a cohort study between British and South Asian infants, South Asian infants have smaller kidneys than British white infants, even after considering potential confounding factors such as birth weight (Roderick et al., 2015).

Psychosocial well-being can be identified as a superordinate construct as it includes emotional or psychological well-being, as well as collective well-being (Eirosa-Orosa, 2020). According to Encyclopedia of the UN Sustainable Development Goals (Kumar, 2020), psychosocial well-being is a multidimensional construct incorporated with the physical, economic, social, mental, emotional, cultural, and spiritual determinants of health. Diener and Diener (1995) pointed out that, subjective well-being, which is comparable to one’s quality of life, pertains to both the cognitive evaluation of life satisfaction and the emotional responses experienced by the individual. He has emphasized that personality traits, income levels, and social support play key roles in shaping the well-being of individuals across cultures. Notably, the way people perceive and experience the factors contributing to their well-being can vary between individualistic and collectivist cultures.

The term “quality of life” (QOL) is similar to psychosocial well-being as it involves emotional, social, and physical components (Eirosa-Orosa, 2020). Further, this term is used in healthcare research to measure how an individual’s well-being is affected by medical conditions over time. Especially, chronic diseases can reduce the life expectancy or the life quality of individuals (Christensen et al., 2004). QOL is not just an abstract or subjective concept but holds tangible relevance in the context of ESRD patients. It implies that how patients perceive their own well-being and daily functioning has a direct impact on their health outcomes, including the risk of developing complications or even death (Kimmel et al., 1998; Kimmel, 2006). ESRD can affect a patient’s health-related quality of life (HRQOL) in many ways. The symptoms, side effects of the medicines, various food and fluid restrictions, limitations related to social life, as well as associated stigma and taboo can negatively affect the well-being of patients with CKD (Senanayake et al., 2020). According to the reports of some patients with advanced CKD, their health-related quality of life was equivalent to those with terminal malignancy (Webster et al., 2017). Ultimately, HRQOL will affect the overall well-being of individuals with chronic kidney disease undergoing hemodialysis.

Therefore, the Kidney Disease Outcomes Quality Initiative (K/DOQI), a leading organization developing standards and guidelines related to CKD, and the Center for Medicare Services in the United States have identified the importance of health-related quality of life of all patients undergoing dialysis treatment (Senanayake et al., 2020).

Furthermore, the prevalence of mental health conditions are much higher among ESRD patients than among patients with other chronic conditions (Chilcot et al., 2010). Depression has been recognized as the most common psychological problem that causes resistance to treatment and there is a significant association between depression and mortality of dialysis patients (Andrade et al., 2010; Ma and Li, 2016). Moreover, Andrade and Sesso (2012) found that the percentage of depressive symptoms among patients undergoing dialysis is slightly higher compared to patients under conservative treatment of CKD. According to the authors, the disease’s physical discomforts and the demands of dialysis treatment can worsen individuals’ functional capacity, potentially resulting in unemployment and a lack of monthly income, ultimately leading to depression. However, individuals undergoing conservative treatments face fewer challenges in comparison to those on hemodialysis.

Moreover, caregivers of patients with advanced CKD who are undergoing dialysis play a pivotal role in the coordination of care, such as medication administration, preparation of a special diet, transportation to the hospital for clinical attendance, dialysis, and personal care (Adejumo et al., 2019). Deterioration of family relationships, stress, and social isolation are frequently encountered by CKD caregivers (Brunier and McKeever, 1993). Low et al. (2008) emphasized the importance of promoting the psychological health of close persons of individuals with ESRD to continue to care effectively. Furthermore, they highlighted the lack of studies regarding how health services support close persons of individuals with ESRD and the necessity of further research to explore the relationship between health services and close persons to empower the interventions of individuals with ESRD.

According to Polish statistics, CKD is a major risk factor for cardiovascular disease (Zdrojewski et al., 2016). A study on a Polish sample of CKD hemodialyzed and non-dialyzed patients revealed that there is a higher rate of depression among hemodialyzed patients (Cwiek et al., 2017). Furthermore, this study highlighted the importance of monitoring the mental state of CKD patients and providing timely psychological care. Another study investigated the prevalence of CKD and its relation to the socioeconomic status among the Polish elderly population. The results indicated that CKD is a frequent disease and affects one-third of the Polish elderly population (Chudek et al., 2013). Additionally, CKD is frequently present among urban residents, non-smokers, alcohol abstainers, less physically active people, and less educated women. A comparative study on the quality of life in patients with ESRD and undergoing hemodialysis revealed that their quality of life is low compared to the control group (Dembowska et al., 2022).

Based on the information provided, CKD can be recognized as a worldwide public health threat that has not yet been adequately tackled (McKercher et al., 2013; Wimalawansa, 2015). In developing countries like Sri Lanka, the need to provide not only accessible healthcare for the patients but also mental health support is urgent. According to the report of the International Expert Consultation on Chronic Kidney Disease of unknown etiology in Sri Lanka (World Health Organization, 2016), developing effective interventions for promoting the well-being of CKD patients was highly recommended. Hence, activities aimed at prevention, early detection, treatment, care, surveillance, and social interventions also were recommended. Even though the Sri Lankan government has implemented the recommended activities to a certain extent for 30 years, CKD is still a tragedy for those who are affected. CKD outcomes already overburden the country’s health sector, and yet there are indications of cutting back on services such as dialysis and kidney transplant (Liyanage, 2015). According to Ranasinghe and Ranasingha (2015) providing psychosocial support to both individuals with CKD and their family members, increasing the allowances, and effectively using all personnel, ministries, and media support are important to eliminate this health hazard from Sri Lankan society. An ethnographic study by Liyanage (2022), it is crucial to conceptual shift to an ethno-medical model to address CKD in Sri Lanka. The author suggested improving the cultural competency and communication skills of healthcare providers to apply the “bio-psychosocial perspective in the healthcare delivery system to bridge the gap between the community and the hospital. Moreover, although the international human rights regime could successfully address the humanitarian needs of chronic diseases like CKD, the domestic jurisdiction is yet not to be prepared to address this fatal disease (Wijayath, 2019).

In our paper, we emphasized that psychosocial well-being is a multifaceted concept often referred to as quality of life. One of the dimensions of quality of life is health-related quality of life (HRQOL), which specifically pertains to the experiences of individuals dealing with chronic illnesses. Nevertheless, quality of life goes beyond just health-related aspects and encompasses various aspects of daily life, including emotional well-being, marital status, income, housing, cultural background, personal values, spirituality, and overall life satisfaction. Kimmel (2006) pointed out that there is still a lack of clear and adequate methods for evaluating the QOL in individuals with CKD and it is essential to establish a standardized framework that specifically addresses QOL assessment, including aspects like depressive affect, the perception of the burden of illness, and social support. Therefore, this study aims to evaluate the quality of life of individuals with ESRD by comprehensively addressing all the mentioned aspects.

Chronic Kidney Disease (CKD) carries stigma due to factors like the need for dialysis, changes in appearance, functional issues causing pain, feelings of constraint, and sexual challenges (Pedreira Robles and Aguayo-González, 2019). These stigmas can lead to feelings of shame and guilt in individuals with CKD. Although there is research on guilt and shame related to chronic illnesses, no studies have explored shame and guilt connected to chronic conditions across both individualistic and collectivist cultures. Therefore, this study addresses the gap in the research literature. Moreover, cultural beliefs and behaviors influence an individual’s perception of disease etiology, illness, treatment, and disease labels (Kleinman et al., 1995; Turner, 1996). Hence, the cultural approach of the patient becomes a significant factor, as it determines the patient’s care-seeking behavior, treatment options, choices, and compliance. Therefore, this study will provide an opportunity to understand how cross-cultural beliefs determine the understanding of the disease and expectations toward individuals with CKD and their caregivers.

Learning from the experience of other countries that are undergoing the process of implementing the WHO/UE CKD treatment standards, such as Poland, can inform the practitioners working with CKD patients in Sri Lanka. However, according to the research literature, the importance of providing comprehensive psychosocial support to patients with advanced CKD and with hemodialysis was highlighted regardless of the socioeconomic status of the country. Thus, assessing ESRD patients’ psychosocial well-being in these two countries and comparing their healthcare systems might enable them to form recommendations for the Sri Lankan health service.

### 1.1 Objectives

The study’s primary objective is to investigate the psychosocial well-being of individuals with chronic kidney disease undergoing hemodialysis and their relatives in Sri Lanka and Poland. Its specific objectives are as follows:

1. To compare the psychosocial well-being among individuals with chronic kidney disease undergoing hemodialysis in Sri Lanka and Poland.
2. To evaluate medical (having diabetes, having hypertension) and social factors (gender, age, marital status) that might be associated with psychosocial well-being of individuals with chronic kidney disease undergoing hemodialysis.
3. To explore how individuals with chronic kidney disease undergoing hemodialysis and their caregivers experience the disease and the process of treatment in Sri Lanka and Poland.
4. To provide guidelines to improve psychosocial well-being among individuals with chronic kidney disease undergoing hemodialysis and their caregivers in Sri Lanka.

### 1.2 Research questions

1. Are there differences In HRQOL, depression, emotional suppression, shame and guilt proneness, and acceptance of illness among individuals with chronic kidney disease undergoing hemodialysis In Sri Lanka and Poland?

2a. Does gender predict variations in HRQOL, depression, emotional suppression, shame and guilt proneness, and acceptance of illness among individuals with chronic kidney disease undergoing hemodialysis?

2b. Does age predict variations in HRQOL, depression, emotional suppression, shame and guilt proneness, and acceptance of illness among individuals with chronic kidney disease undergoing hemodialysis?

2c. Does marital status predict variations in HRQOL, depression, emotional suppression, shame and guilt proneness, and acceptance of illness among individuals with chronic kidney disease undergoing hemodialysis?

2d. Does having diabetes predict variations in HRQOL, depression, emotional suppression, shame and guilt proneness, acceptance of illness among individuals with chronic kidney disease undergoing hemodialysis?

2e. Does having hypertension predict variations in HRQOL, depression, emotional suppression, shame and guilt proneness and acceptance of illness among individuals with chronic kidney disease undergoing hemodialysis?

3a. How do patients with CKD experience the disease and process of treatment?

3b. How do the caregivers of individuals with chronic kidney disease undergoing hemodialysis experience the disease and process of treatment?

We hypothesized that there are differences in psychosocial well-being (operationalized as health-related quality of life, depression, shame and guilt-proneness, emotional suppression, and acceptance of the illness) among individuals with chronic kidney disease undergoing hemodialysis in Sri Lanka and Poland. As CKD affects populations in different regions of the world unequally and as a result of their demographic characteristics, comorbidities, and access to healthcare resources (Kovesdy, 2021), we presume that there will be group differences in psychosocial well-being. Limited research has delved into the notion of acceptance of illness within the context of Chronic Kidney Disease (CKD), despite the considerable focus on other chronic illnesses (Chan, 2013). Notably, the degree of acceptance appears to be more pronounced in recipients of kidney transplants in comparison to individuals undergoing dialysis. Moreover, this acceptance level is linked to factors like age, ethnicity, and even instances of transplant failure (Keogh and Feehally, 1999). Stage (2022) investigated the intricate impact of shame on individuals with chronic conditions, particularly through storytelling. The study revealed that shame is connected to feeling tired and sluggish and shaped by cultural and political factors, which challenges the notion of solely concentrating on personal health enhancement. Moreover, Sharing stories of shame among peers on social media can help alleviate this pressure. Stage concluded that the experience of shame related to chronic conditions is not well-studied, and the study provides a starting point for understanding this topic.

In addition, we assumed that gender, age, marital status, diabetes, and hypertension predict the psychosocial well-being of individuals with chronic kidney disease undergoing hemodialysis. According to previous studies, the prevalence of CKD is higher among females than males (Kafle et al., 2019; Kovesdy, 2021). Some studies pointed out the gender disparities in the quality of life of CKD patients. Mujais et al. (2009) found that the female gender was a significant predictor of low HRQOL in CKD patients. Similarly, Paraskevi (2011) reported female patients who have ESRD are more depressed than male patients. However, another study confirmed that both male and female patients presented with high levels of depression compared to the control group (Fountoulakis et al., 2001). However, Senanayake et al. (2020) found that gender was not a significant predictor of HRQOL. Kovesdy (2021), mentioned that the prevalence of CKD increased with advancing age. Paraskevi (2011), reported that younger CKD patients have better QOL in physical, psychological, and social well-being and that the older patients are falling behind in social activities and interests, being more socially restricted and depressed.

Concerning marital status, previous studies note it as a protective factor for CKD patients’ QOL. Marital status is positively associated with the good quality of life of patients with ESRD (Gerogianni and Babatsikou, 2014a, b). Furthermore, Paraskevi (2011) found that compared to divorced CKD patients, married patients enjoy a better quality of life and are satisfied in their lives. However, according to John and Thomas (2012), intimate relationships and sexual activities among couples are badly affected by ESRD.

Mujais et al. (2009) found that the HRQOL of CKD patients is not only influenced by the stage of the CKD, age, and gender, but also by having diabetes. According to Megari (2013) and Anees et al. (2011) having diabetes and experiencing its complications is directly associated with decreased quality of life. Comparably, Senanayake et al. (2020) reported that the advanced stage of CKD and being diagnosed with depression were significantly associated with low HRQOL. Andrade and Sesso (2012) found that depression was associated with some clinical and sociodemographic variables. However, Andrade et al. (2010) reported that there was no significant difference between the stages of the disease. Last but not least, Soni et al. (2010) highlighted the importance of controlling hypertension for better HRQOL. Thus, we presume that having diabetes and hypertension will predict CKD patients’ psychosocial well-being and depression.

## 2 Methods

### 2.1 Study design

The project consists of two phases and assumes a mixed-method study design to deeply investigate the psychosocial well-being of individuals with chronic kidney disease undergoing hemodialysis and their caregivers. Hence, quantitative and qualitative methods will be combined. The quantitative research will evaluate the psychosocial well-being of individuals with chronic kidney disease undergoing hemodialysis by measuring their HRQOL and levels of depression as psychosocial well-being is a multifaceted concept. The qualitative study will focus experiences of individuals with chronic kidney disease undergoing hemodialysis and their caregivers to address the areas that cannot be assessed with quantitative study (e.g., thick description of perceptions, knowledge, beliefs, emotions, social, and medical support of individuals with chronic kidney disease undergoing hemodialysis and their caregivers which addresses the psychosocial wellbeing). There are several ways of mixing quantitative and qualitative methods. In this study, qualitative and quantitative data will be collected and analyzed simultaneously. Then the results of both studies will be integrated and discussed in view of the main objective of the study (Adoli et al., 2022). The methodologies of the quantitative and qualitative studies and the strategies for integration of the results will be discussed in the next sections.

The quantitative data (phase 1 of this project) will be collected in a cross-sectional study and analyzed with SPSS 25 software. Regression models and dependent group differences will be tested. The qualitative data (phase 2 of the project) will be collected using semi-structured interviews and analyzed with qualitative content analysis (Table 1).

**Table 1:** Summary of study design.

Phase	Group 1 (Sri Lankan)	Group 2 (Polish)	Method	Instruments	Study design	Data analysis
1	63 patients	63 patients	Quantitative	Kidney Disease Quality of Life-Short Form (KDQOL-SF) Version 1.3 Beck Depression Inventory Test of Self-Conscious Affect (TOSCA-3) Courtauld Emotional Control Scale (CECS) Acceptance of Illness Scale (AIS)	Cross-sectional design	Statistical analysis: regression models, testing differences between two samples (t-test for two independent samples or U Mann–Whitney test)
2	The sample size is not pre-determined beforehand	The sample size is not pre-determined beforehand	Qualitative	Semi-structured interviews	Cross-sectional design	Qualitative content analysis

The project’s initial phase aims to enroll a total of a minimum 126 individuals with chronic kidney disease undergoing hemodialysis undergoing dialysis treatment, with at least 63 participants from each country (see for reference Kohn and Senyak, 2021). The selection of this sample size takes into account practical considerations related to the clinical population. Additionally, a matching sampling technique will be employed specifically for the Polish group, which will enhance the study’s validity. Although larger sample sizes might be preferred, the chosen number is the most feasible and suitable given the study’s objectives and limitations. Sample size bias will be taken into account while processing the data and making conclusions while at the same time, we are mindful of resource limitations and the unique characteristics of our study population.

Randomly selected individuals with chronic kidney disease undergoing hemodialysis who participated in the first phase of the study and their caregivers will participate in the second phase (qualitative part) of the study from each country. However, the specific number of participants for this phase will not be predetermined, as determining the sample size in advance is inherently challenging due to the exploratory nature of this study. The data saturation will determine the sample size.

To achieve the target sample size, participants will be recruited at the hemodialysis units of the hospitals. Once the research proposal receives approval from relevant ethical committees in both countries, the field arrangements will be finalized. Data collection will commence only after obtaining all the necessary permissions and consent of the participants and relevant personnel.

### 2.2 Study setting

The project will be held in two countries (Poland and Sri Lanka) in two hemodialysis units of the following hospitals based on a purposive sampling design: the Hemodialysis Unit of the District Teaching Hospital, Kandy, Sri Lanka, and the Hemodialysis Unit of The University Clinical Center of the Medical University of Gdańsk, Gdańsk, Poland. Kandy Hospital is planned to choose as the Sri Lankan study location as it lies in the central part of Sri Lanka and patients from different parts of the country can easily access this hospital. Therefore, it is easily accessible for the patients. The University Clinical Center of the Medical University of Gdańsk is planned to participate in the study as this is the only Polish Nephrology Clinic for adults that is part of the European Reference Network for Rare Kidney Diseases.

### 2.3 Eligibility criteria

The study participants with CKD will be recruited according to the following eligibility criteria:

1. being a person who is diagnosed with stage V of CKD, as patients whose CKD advancement is from stages I to IV are not undergoing dialysis treatment;
2. being a person who is undergoing hemodialysis treatment;
3. being within the age range from 18 to 70 years;
4. being a person whose CKD treatment has been lasting for more than 6 months as this time duration might bring a significant burden for patients.
5. the study has also nationality inclusion criteria, which are Sinhala for the Sri Lankan sample and Polish for the Polish sample.
6. comorbidities will not be considered for inclusions.

The exclusion criteria for patients include:

1. Persons who do not meet the defined inclusion criteria,
2. Persons who decline to provide consent.
3. Persons who lack the physical or mental capability to participate in the study.

The inclusion criteria for caregivers include:

1. Caregivers (who can be related or non-related, e.g., friend, spouse, child, sibling, or parent) of CKD patients will be eligible if they are identified by the participating CKD individuals as their significant supporters.
2. Caregivers who are over 18 years old.

The exclusion criteria for caregivers include:

1. Caregivers who do not meet the defined inclusion criteria.
2. Caregivers who refuse to give consent.
3. Caregivers who lack the physical or mental capacity to participate in the study.

### 2.4 Recruitment

Individuals with chronic kidney disease undergoing hemodialysis who will meet the eligibility criteria will be enrolled at the hemodialysis unit at the District Teaching Hospital in Kandy (Sri Lanka) and the hemodialysis unit at the University Clinical Center of the Medical University of Gdansk in Gdansk (Poland) by a researcher through medical records. The researcher will be responsible for the first contact with potential participants in order to provide them with oral and written information about the project, alongside an informed consent form to sign. Participation in the project will be voluntary. Each participant will have the right to refuse to participate in the study without any negative impact on the care received in the hospital.

Moreover, the participants will be assured of the confidentiality of their interviews and the subsequent data arising from the same. The anonymity of the participants will be protected by all means, for which they will be given pseudonyms/numbers and any information that could reveal their identity will be wiped out from the transcripts. They will be asked to reach out to the researchers if they felt any discomfort during and after the interviews.

### 2.5 Variables and measurement tools

There are five dependent variables in this study - health-related quality of life, depression, shame and guilt proneness, emotional suppression, and acceptance of illness.

#### 2.5.1 Health-related quality of life

Kidney Disease Quality of Life (KDQOL-SF) Version 1.3 will be used to assess the health-related quality of life of individuals with chronic kidney disease undergoing hemodialysis. KDQOL-SF questionnaire is a self-report measure developed by Hays et al. (1997) for individuals with kidney disease and on dialysis. This instrument has good construct validity and test–retest reliability (Senanayake et al., 2020). KDQOL has two components; Kidney Disease Specific Component and SF-36. Altogether, the instrument has 81 questions in 19 domains. 11 domains assess kidney disease-specific components through 43 questions and 8 domains assess the general health-related components through 36 questions. The 11 domains of Kidney Disease Specific Components are symptom/problem list (12 items), the effect of kidney disease on daily life (8 items), the burden of kidney disease (4 items), cognitive function (3 items), quality of social interaction (3 items), sexual function (2 items), sleep (4 items), social support (2 items), work status (2 items), patient satisfaction (1 item) and dialysis staff encouragement (2 items) (Senanayake et al., 2017).

SF-36 components are physical function (10 items), role limitations caused by physical problems (4 items), role limitations caused by emotional problems (3 items), pain (2 items), general health perceptions (5 items), social function (2 items), emotional well-being (5 items), and energy/fatigue (4 items). The final item assesses the overall health on a scale from 0 to 10. Different questions have different answer options, which range from two to seven.

When scoring, each question is scored on a scale ranging from 0 (worst health) to 100 (best health). Three summary scores Kidney Disease summary component (KDSC), Physical Component Summary (PCS), and Mental Component Summary (MCS) will be derived from 19 domain scores of KDQOL-SF, by averaging the domain scores in respective three summary scores. Summary scores range from 0 to 100 and a higher score will represent better Health-Related Quality of Life (Senanayake et al., 2020). The KDQOL-SF was culturally validated and adapted for Sri Lankan (Senanayake et al., 2017) and Polish (Sapilak et al., 2006) cultural settings.

#### 2.5.2 Depression

Beck Depression Inventory (BDI) will be used to assess the depression of individuals with chronic kidney disease undergoing hemodialysis. It is a 21-item, self-administered questionnaire that covers the full spectrum of depressive symptomatology (Bautovich et al., 2018). Those items cover areas such as 1. Mood, 2. Pessimism, 3. Sense of Failure, 4. Lack of satisfaction, 5. Guilt Feeling, 6. Sense of Punishment, 7. Self-dislike 8. Self-accusation, 9. Suicidal Wishes, 10. Crying, 11. Irritability, 12. Social Withdrawal, 13. Indecisiveness, 14. Distortion of Body Image, 15. Work Inhibition, 16. Sleep Disturbance, 17. Fatigability, 18. Loss of Appetite, 19. Weight Loss, 20. Somatic Preoccupation, and 21. Loss of Libido (Beck et al., 1988).

The items are rated on a 4 -point Likert scale (0–3) and ranked for severity within the time frame of the past 2 weeks and the total score will be obtained by adding the values of the selected sentences. The range of the score that can be obtained is 0–63 points. Cut-off score guidelines for the BDI-II are given with the recommendation that thresholds be adjusted based on the characteristics of the sample, and the purpose for use of the BDI-II. A total score of 0–13 is considered a minimal range, 14–19 is mild, 20–28 is moderate, and 29–63 is severe (Jackson-Koku, 2016).

The BDI has a reliability measure (Cronbach’s alpha) of 0.87 (González-Flores et al., 2021) Some items can be shifted as per the population sample, and those items are called affective or emotional items (Arnarson et al., 2008). Furthermore, the BDI has been frequently used to assess depression in patients with end-stage renal disease (Richter et al., 1998; Andrade and Sesso, 2012). In the Polish cultural setting, the Polish adaptation of BDI-II will be used (Zawadzki et al., 2009). In the Sri Lankan cultural setting, the BDI - II adaptation prepared by Rodrigo et al. (2015) will be used.

#### 2.5.3 Shame and guilt proneness

The Test of Self-Conscious Affect (TOSCA-3) questionnaire will be used to assess shame and guilt proneness of individuals with chronic kidney disease undergoing hemodialysis. This questionnaire was developed by Tangney et al. (1989) (Broerman, 2020). This is a tool that employs brief scenarios to evaluate individuals’ tendencies to experience shame and guilt. These scenarios reflect everyday situations, like someone breaking something at work and hiding it. Respondents choose from different options that represent either shame or guilt. For instance, one option might be thinking about leaving the job (related to shame), while another could be feeling bothered and wanting to fix the issue (related to guilt). Respondents rate how likely they had choose each response on a 5-point scale. This helps them show if they would feel both shame and guilt in each situation.

The questionnaire includes 11 negative and five positive scenarios. Respondents rate their responses from 1 to 5. These ratings determine scores on six scales: guilt-proneness, shame proneness, externalization (blaming others), indifference to responsibility, pride in oneself (alpha pride), and pride in behavior (beta pride). For each scenario, respondents show how likely each reaction is, usually ranging from 4 to 5. A score of 1 means unlikely, while 5 means very likely. In our study, we will utilize the Polish adaptation of the test (Adamczyk and Sobolewski, 2022). It demonstrates good consistency in its results over time. The reliability for shame measurement was 0.85 over 3 months, and for guilt measurement, it was 0.74 over 5 months (Adamczyk and Sobolewski, 2022).

#### 2.5.4 Emotional suppression

The Courtauld Emotional Control Scale (CECS) will be utilized to evaluate the emotional control of individuals with chronic kidney disease undergoing hemodialysis. This primary assessment tool is widely employed to measure the subjective management of negative emotions such as anger, anxiety, and depression in challenging situations. Designed to encompass both healthy and unwell adults, CECS was originally developed by Watson and Greer in 1983 (Lewicka et al., 2012). The CECS comprises 21 items, organized into three subcategories focusing on suppressing or expressing anger, anxiety, and depressed mood. Respondents rate their agreement with item statements on a 4-point scale that ranges from “Almost never” to “Almost always.” The items are scored in such a way that higher scores indicate greater control over emotional responses. The different parts of the scale showed good internal consistency, with reliability scores ranging from 0.86 for the anger section to 0.88 for the sections measuring depressed mood and anxiety. The connections between these sections and the total scores suggest that the questionnaire effectively gauges how people generally handle their emotions. When tested again after three to 4 weeks (with a group of 40 participants), the scale’s reliability remained strong: 0.86 for anger, 0.84 for anxiety, 0.89 for depressed mood, and an impressive 0.95 for the overall CECS score (Durá et al., 2010).

#### 2.5.5 Acceptance of illness

As quality of life is interrelated with factors like pain and the degree of acceptance of an individual’s illness, we decided to assess for acceptance of illness in individuals with chronic kidney disease undergoing hemodialysis. It will be measured through the utilization of the Acceptance of Illness Scale (AIS), introduced in 1984 by B. J. Felton, T. A. Revenson, and G. A. Hinrichsen (Polish adaptation by Czerw et al., 2021). The AIS questionnaire comprises eight statements that outline adverse outcomes of compromised health, encompassing evaluations of limitations imposed by the illness, reduced self-sufficiency, a sense of dependence on others, and diminished self-esteem. The overall assessment of illness acceptance is calculated as the cumulative score of all the statement points. A lower score signifies a lack of adaptation to the illness and a strong sense of psychological discomfort, while a higher score indicates acceptance of one’s medical condition and is accompanied by a dearth of negative emotions tied to the illness. Each statement presents a five-point scale, with patients indicating their current health status by selecting the appropriate number: 1 – Strongly agree, 2 – Agree, 3 – Uncertain, 4 – Disagree, 5 – Strongly disagree. Choosing answer number 1 denotes poor adaptation to the disease, while answer number 5 indicates complete acceptance of the disease. The sum of all points falls within a range of 8 to 40, serving as a measure of illness acceptance. The AIS scale exhibits the reliability of Cronbach’s α at 0.83 (Felton et al., 1984).

Apart from the instruments mentioned above, a sociodemographic survey will be distributed to all study participants (both individuals with chronic kidney disease undergoing hemodialysis and their caregivers). The survey will measure independent variables. The following information will be collected:

- *Nationality*: Participants’ nationality will be the grouping variable. Information on the participant’s nationality will be gathered with an open question in the demographic information sheet. The question will be: “Please, state your nationality.” The participants will write down their answers.
- *Gender:* Information on the participant’s gender will be collected using a single-choice question. As gender will be considered bivariate, the participants will mark if they are a woman or a man.
- *Age:* The participant’s age will be measured as a continuous variable. Thus, the participants will be asked an open question “How old are you?,” the participants will write down their answers.
- *Ethnicity:* Information on the participant’s ethnicity will be collected using a single-choice question. The participants will be asked an open question “What is your ethnicity?,” the participants will write down their answers.
- *Religion*: Information on the participant’s religion will be collected using a single-choice question. The participants will be asked an open question “What is your religion?,” the participants will write down their answers.
- *Marital status*: Information on the participant’s marital status will be collected using a multiple-choice question. The participants will be able to choose among options such as: married, widowed, separated, divorced, single, or in an informal relationship. The participants will mark their answers.
- *Educational level*: Information on the participant’s education level will be collected using a single-choice question. The participants will be asked to answer the question about the last level of education they earned. They will be presented with options such as: primary, junior secondary, senior secondary, collegiate, and tertiary. The participants will mark their answers.
- *Occupation*: Information on the participant’s profession will be collected using an open question (“What is your profession?”). The participants will write down their answers. (This question will be asked from the individuals with chronic kidney disease undergoing hemodialysis only)
- *The stage of CKD:* (self-reported; considered as a number from 1 to 5) will be asked using an open-ended question (What is your stage of CKD?/What is your friend’s/relative’s stage of CKD?). The participants will write down their answers.
- *Diabetes* (self-reported): the information about this will be collected by asking an open-ended question (“Do you have diabetes?”) and the participant will write down the answer (This question will be asked from the individuals with chronic kidney disease undergoing hemodialysis only).
- *Hypertension* (self-reported):the information about this will be collected by asking an open-ended question(Are you suffering from Hypertension?) and the participant will write down the answer (This question will be asked from the individuals with chronic kidney disease undergoing hemodialysis only).
- *Cardiovascular disease* (self-reported): the information about this will be collected by asking an open-ended question(Are you suffering from Cardiovascular disease?) and the participant will write down the answer (This question will be asked from the individuals with chronic kidney disease undergoing hemodialysis only).
- *The number of family members diagnosed with CKD*: Information on the number of family members diagnosed with CKD will be gathered with an open question in the demographic information sheet (“How many members of the family are diagnosed with CKD?”). The study participants will note the number.
- *The history of the disease/the duration* - the information about this will be collected by asking an open-ended question (How long have you had this disease?) and the participant will write down the answer.
- *The information about the relationship to the patient* will be asked from caregivers.

The objective of the second phase of the study is to explore the experiences of the disease of individuals with chronic kidney disease undergoing hemodialysis and their caregivers in Sri Lanka and Poland with the aim of a deeper understanding of the psychosocial well-being of the target group. The qualitative part of our research does not have a predetermined sample size, as we recognize the challenges associated with setting one in exploratory studies (Emmel, 2013; Sim et al., 2018). Our main focus in this phase is to explore the experiences of individuals with chronic kidney disease undergoing hemodialysis and their caregivers to gain a comprehensive understanding of the complex concept of psychosocial well-being within this target group. Our approach is to conduct a broad study of the participants’ experiences, allowing us to embrace and uncover key themes that may emerge during the research process, rather than imposing predetermined notions. Although we anticipate conducting a smaller number of interviews, our goal is to attain robust qualitative insights into the psychosocial well-being of individuals with chronic kidney disease undergoing hemodialysis and their caregivers (Adler and Adler, 2012). This thoughtful methodology will enable us to deeply understand the psychosocial well-being of this specific population.

The interviews will be based on semi-structured interview guides. There will be two semi-structured interview schedules – one for individuals with chronic kidney disease undergoing hemodialysis and one for their caregivers. Each interview guide consists of 27 questions. The first 12 questions will be based on general information about both patients and caregivers. The rest of the questions will be open-ended and allow participants to express their answers in detail. The open-ended questions will focus on patients’ and caregivers’ experiences, emotions, perceptions, relationships, social support, and medical support they receive. The questions in Sinhala and Polish languages are presented in Attachment 1 and Attachment 2, respectively.

While the study intends to include both patients and their caregivers (caregivers), the interviews will be conducted separately for each participant. Nonetheless, the subsamples are interconnected, allowing for future dyadic analysis. The sample size for each of the subsamples will be determined by data saturation.

The interviews will be audio recorded and will be literally and systematically transcribed. Identifying data of the informants in the transcripts will be anonymized. Moreover, images capturing significant psychosocial factors and elements concerning CKDu patients will be captured, following the hospital’s approval and ensuring that no specific patients are depicted.

### 2.6 Design and method

Semi-structured interviews with the first group (Sri Lankan) will be conducted by the principal investigator (PI) at the Hemodialysis unit of the Kandy Hospital, Sri Lanka. The semi-structured interviews with the second group (Polish) will be conducted by trained research assistants at the Hemodialysis unit of The University Clinical Center of the Medical University of Gdansk, Gdańsk, Poland. As a Sri Lankan Ph.D. student, the PI is not familiar with the Polish language. Therefore, MA students from the Institute of Psychology, University of Gdansk, will be recruited and trained for conducting interviews. The psychosocial approach to understanding the experience of CKD is a novel area of research. Thus, research assistants will be given an informed orientation into the research project and proper training in its methods before they enter the field.

### 2.7 Data management

The raw quantitative data will be entered into Microsoft Excel format to allow for statistical analysis via SPSS. Qualitative data will be stored on multiple hard drives such as USB and Google Drive and manual copies such as files and notebooks. Only researchers who belong to the project will have access to data and the data will be locked in a safe storage box at the university. However, the datasets and/or analyses related to the current study will be available from the corresponding author upon reasonable request.

### 2.8 Bias

As the data will be collected from hemodialysis units of the hospitals where the patients will be undergoing the procedure, some of them might have difficulties answering the questionnaires by themselves. On such occasions, the researcher will be reading the questions to the study participant and mark their answers on the paper questionnaire. This may lead to response bias. To minimize this effect, patients will be ensured of their anonymity. Furthermore, questions and answers options will be read to patients very clearly and repeated if not heard clearly.

## 3 Data analysis

### 3.1 Overview

The quantitative data obtained from the validated questionnaires (i.e., KDQOL-SF and BDI) will be analyzed with SPSS 25 (Statistical Package for Social Science). The variables will be computed using relevant scoring methods. Due to the sample size limitation, non-parametric methods will be employed (Altman and Bland, 2009).

The qualitative data will be analyzed using a conventional qualitative content analysis approach (Cho and Lee, 2014). The goal of this approach is to identify frequently reported themes/codes emerging from the data. Therefore, all the responses will be read multiple times as the first step of the analysis and an initial list of themes will be prepared by each graduate student. After that, each of the prepared separate lists of themes will be discussed, and a final list of themes (based on the most commonly reported themes) will be prepared. Finally, these themes will be analyzed further to identify emerging patterns that will finally lead to a deeper understanding of the psychosocial well-being of CKD patients and their caregivers.

### 3.2 Integration

The results of the quantitative and qualitative studies will be integrated by looking at common concepts and how qualitative data provide a deeper understanding of quantitative data. For instance, the KDQOL scale may address whether individuals with chronic kidney disease undergoing hemodialysis are affected by the disease by means of work or other regular activities and relationships. Participants are supposed to give answers as “Yes” or “No”; which will be not provided sufficient information about the experiences of individuals with chronic kidney disease undergoing hemodialysis. However, qualitative data will cover up the gap in the information as questions such as “How do you understand CKD?” and “How has your situation shaped your day-to-day life?” will allow participants to give in-depth descriptions of their experiences of the disease. Furthermore, while BDI screens the symptoms of depression, qualitative data may provide an in-depth understanding of individuals’ suffering by asking open-ended questions about their feelings. Therefore, by integrating both quantitative and qualitative data, it is expected to be enriched the data about the psychosocial well-being of individuals with chronic kidney disease undergoing hemodialysis and their caregivers.

### 3.3 Status and timeline of the study

The study is in the recruitment and data collection stage. Ethical approval for the study with group 1 (Sri Lankan sample) has been obtained from the Ethics Review Committee of the Open University Sri Lanka and ethical approval for group 2 (Polish sample) was obtained from the Ethics Committee of the University of Gdansk. Study instruments were finalized and data collection commenced in mid-October 2022, to be completed in September 2023.

## 4 Discussion

### 4.1 General discussion

The proposed study aims to focus on the psychosocial well-being of individuals with chronic kidney disease undergoing hemodialysis and their caregivers by using both quantitative and qualitative methods. Validated questionnaires and semi-structured interview guides will be employed for data collection.

The primary goal of this study is to explore and investigate the psychosocial well-being of CKD patients and their caregivers in both Sri Lanka and Poland. The knowledge gained from this research will be invaluable in promoting a holistic and systemic approach to planning, prevention, and intervention strategies tailored to the needs of CKD patients. By understanding the psychosocial well-being of this population, we can work toward enhancing their overall quality of life and well-being.

In line with the study’s aim, we are committed to providing necessary support for participants identified as requiring psychological assistance. Any Sri Lankan patients in need will be referred to mental health services, ensuring that they receive the appropriate care and support during their journey with CKD.

Patients from Poland who participate in the study will be appreciated for their involvement, as they will receive written feedback on the project via email after the study concludes. This gesture ensures transparency and gratitude for their contribution to advancing research in this field.

A distinctive aspect of this study lies in its contribution to the research literature. While previous studies have explored the quality of life of ESRD patients, our research takes a step further by investigating the broader concept of psychosocial well-being using a mixed-method approach. By comparing the psychosocial well-being of two different ethnicities, Sri Lankan and Polish, we aim to uncover the influence of culture and ethnicity on the experiences of individuals with chronic kidney disease undergoing hemodialysis and their caregivers.

By delving into the factors associated with culture and ethnicity, we seek to gain a deeper understanding of how these elements shape the psychosocial well-being of the target group. This knowledge can inform more culturally sensitive and tailored approaches to support and care for CKD patients in both Sri Lanka and Poland.

Ultimately, the findings of this study will contribute to the development of specific guidelines for hospitals and clinics responsible for treating CKD patients. By considering the psychosocial aspects of CKD care and incorporating the insights gained from this research, healthcare facilities can improve their practices, ensuring that patients and their caregivers receive comprehensive and compassionate care throughout their journey with CKD.

In conclusion, this study aspires to make a significant contribution to the field of CKD research by exploring the psychosocial well-being of patients and their caregivers in Sri Lanka and Poland. By adopting a mixed-method approach and comparing different ethnicities, we seek to enrich our understanding of the cultural influences on psychosocial well-being. The ultimate aim is to use these findings to create guidelines that prioritize the holistic care and support of CKD patients, paving the way for better outcomes and improved well-being within this vulnerable population.

The findings will help to explore and investigate the psychosocial well-being of CKD patients and their caregivers in Sri Lanka and Poland. Gathering such knowledge will contribute to promoting a holistic and systemic approach to planning, prevention, and intervention strategies focused on CKD patients. Moreover, the findings of the study will help to enhance the psychosocial well-being of CKD patients and their caregivers. In addition, if during the study some Sri Lankan patients will be identified as in need of psychological support, they will be referred to mental health services (because the aim is to provide the guidelines). Patients from Poland will receive written feedback on the project via e-mail after the study ends (for those who will provide e-mail addresses).

### 4.2 Expected problems

Given that the proposed study is the first of its kind in the Sri Lankan context, it is likely that patients may have questions about the purpose and value of this project. An information sheet detailing the objectives and justification of the research will be prepared and handed over to the participants. The same information sheet will be prepared for the Polish participants.

In addition, prior to the study procedures, the project will be introduced to all participants, allowing them protected time to ask questions and clarify any doubts. It is essential to note that in the Sri Lankan context, CKD patients coming from families with low income receive monthly financial support from the government, along with occasional assistance from certain NGOs. Consequently, participants from these backgrounds might have specific expectations that the research study could offer similar financial benefits. To manage these expectations, it will be explicitly communicated at the beginning of the research that there will not be any direct monetary or material benefit associated with participation. Instead, the benefits lie in their contribution to enhancing the existing knowledge base on CKD. This research aims to have a significant impact on the psychosocial well-being of CKD patients and their families.

Another important problem is the potential of our sample being underpowered due to its size. The choice of minimum sample size takes into account the practical constraints associated with recruiting and managing patients in a clinical setting in two countries. We would like to acknowledge being aware of study limitations related to the sample size. First, we will use non-parametric tests to analyze the data, and what is a good practice while working with small samples (Altman and Bland, 2009). Second, sample size bias will be taken into account while processing the data and making conclusions while at the same time, we are mindful of resource limitations and the unique characteristics of our study population.

### 4.3 Ethical approval

The study proposal has been submitted to the Ethical Review Committee of the Open University of Sri Lanka and received the ethical clearance letter under the application number ER/2022/007 to conduct the first stage of the project. The approval from the Ethical Review Board of the Institute of Psychology, University of Gdańsk, has been obtained under inquiry number 03/2023 for conducting the second stage of the project in Poland.

The research will commence only with the informed consent of the informants to participate in the research project. Participants will be introduced to the research project and they will be offered an opportunity to raise any questions they have about the project before seeking their consensus. They will be provided with an information sheet with a description of the study. The participants will be allowed to withdraw from the study at any point of time during the research period, In case one wishes to withdraw, they will be asked to inform the principal investigator about it. Finally, the data received from the research will be kept in a locked filing cupboard and will be destroyed once the findings are ready to be published.

## Ethics statement

The studies involving humans were approved by Ethical Review Committee of the Open University of Sri Lanka and Ethical Review Board of the Institute of Psychology, University of Gdańsk. The studies were conducted in accordance with the local legislation and institutional requirements. Written informed consent for participation in this study was provided by the participants’ legal guardians/next of kin.

## Author contributions

DB: study conceptualization, study design, data collection, statistical and qualitative analysis, and manuscript drafting. JB: study design, statistical analysis, and manuscript revision. AR: qualitative analysis and manuscript revision. MB: study conceptualization and design, data collection, manuscript revision, supervision, and mentorship. All authors contributed to the article and approved the submitted version.
