# Supplementary material for: Psychosocial well-being among individuals with chronic kidney disease undergoing hemodialysis treatment and their caregivers: a protocol of a mixed method study in Sri Lanka and Poland
Source: Front Psychol. 2023 Dec 7;14:1194991. doi: 10.3389/fpsyg.2023.1194991 (PMC10740214; doi:10.3389/fpsyg.2023.1194991)
Supplement: Supplementary file 1 [file Presentation_1.PDF]

PSYCHOSPOŁECZNY DOBROSTAN I DEPRESJA WŚRÓD PACJENTÓW Z PRZEWLEKŁĄ CHOROBĄ NEREK I ICH BLISKICH KREWNYCH:
BADANIE METODĄ MIESZANĄ NA SRI LANCE I W POLSCE

A. Wywiad częściowo ustrukturowany (dla pacjentów)

Informacje ogólne:

1. Nr badanego

2. Płeć

3. Wiek

5. Rasa

6. Religia

7. Stan cywilny:

zamężna/żonaty; wdowa/wdowiec; w separacji; rozwiedziona/y; kawaler/panna; w związku nieformalnym

8. Wykształcenie

podstawowe, gimnazjum, średnie, wyższe licencjackie/inżynierskie, magisterskie, dr i więcej

Informacje dot. PChN:

10. U ilu osób z rodziny zdiagnozowano przewlekła chorobę nerek (PChN)?

11. Stopień PChN

12. Jak długo chorujesz na PChN (tj. na linii czasu)?

13. Co czujesz po diagnozie PChN ?

14. Jak rozumiesz tę chorobę PChN?

15. Jak Twoja sytuacja zdrowotna wpłynęła na Twoje codzienne życie (odżywianie się, sen, pracę, zachowania, itp.)?

16. Co się stało z twoimi relacjami z rodziną po postawieniu diagnozy PChN?

17. Jak opisałbyś reakcję członków swojej rodziny na PChN?

18. Jak twój stan zdrowia wpływa na twoją rodzinę? (ekonomię, zdrowie, edukacja i relacje itp.)

19. W jaki sposób twoi krewni, przyjaciele i społeczność wspierają cię w przezwyciężaniu Trudności związanych z PChN?

20. Jakie są rodzaje kompetencji osobistych, z których korzystasz w kontakcie z PChN?

21. Jak opisałbyś środowisko medyczne, które Cię leczy ?

22. Jako pacjent z CKD, w jakich aktywnościach społecznych uczestniczysz?

23. Jakie masz zasoby (osobiste, społeczne, kulturowe, medyczne, ekonomiczne), które są pomocne w radzeniu sobie z PChN?

24. Jak myślisz, w jaki sposób religia i rytuały kulturowe pomagają radzić sobie z PChN?

25. W jaki sposób rząd pomaga ci radzić sobie z przewlekłą chorobą nerek?

26. Jakie są wyzwania związane z radzeniem sobie z przewlekłą chorobą nerek?

27. Co należy poprawić, aby lepiej pomagać pacjentom z PChN?

B. Wywiad częściowo ustrukturyzowany (dla krewnych i rodziny)

Informacje ogólne:

1. Nr badanego

2. Płeć

3. Wiek

5. Rasa

6. Religia

7. Stan cywilny:

zamężna/żonaty; wdowa/wdowiec; w separacji; rozwiedziona/y; kawaler/panna; w związku nieformalnym

8. Wykształcenie:

podstawowe, gimnazjum, średnie, wyższe licencjackie/inżynierskie, magisterskie, dr i więcej

9. Pokrewieństwo z pacjentem/stosunek do pacjenta:

Informacje dot. PChN bliskiej osoby:

10. U ilu osób z rodziny zdiagnozowano przewlekła chorobę nerek (PChN)?

11. Stopień PChN u pacjenta

12. Jak dawno u Ciebie/członka Twojej rodziny zdiagnozowano PChN (tj. na linii czasu)?

13. Co czujesz po diagnozie PChN jaką postawiono Twemu członkowi rodziny?

14. Jak rozumiesz tę chorobę PChN?

15. Jak sytuacja zdrowotna Twego członka rodziny wpłynęła na Twoje codzienne życie (odżywianie się, sen, pracę, zachowania, itp.)?

16. Co się stało z twoją relacją z osobą chorą po postawieniu diagnozy PChN?

17. Jak opisałbyś reakcję członków swojej rodziny na PChN?

18. Jak stan zdrowia osoby z PChN wpływa na twoją rodzinę? (ekonomię, zdrowie, edukacja i

relacje itp.)

19. W jaki sposób twoi krewni, przyjaciele i społeczność wspierają cię w przezwyciężaniu

Trudności związanych z chorym z PChN?

20. Jakie są rodzaje kompetencji osobistych, z których korzystasz w kontakcie z chorym z PChN?

21. Jak opisałbyś środowisko medyczne, które Cię leczy Twego krewnego?

22. Jako opiekun chorego z PChN, w jakich aktywnościach społecznych uczestniczysz?

23. Jakie masz zasoby (osobiste, społeczne, kulturowe, medyczne, ekonomiczne), które są pomocne w radzeniu sobie z chorym z PChN?

24. Jak myślisz, w jaki sposób religia i rytuały kulturowe pomagają radzić sobie ze stanem pacjentów z PChN ?

25. W jaki sposób państwo pomaga w radzeniu sobie z przewlekłą chorobą nerek

26. Jakie są wyzwania związane z radzeniem sobie z przewlekłą chorobą nerek?

27. Co należy poprawić, aby lepiej pomagać pacjentom z PChN?
