# Supplementary material for: Psychosocial well-being among individuals with chronic kidney disease undergoing hemodialysis treatment and their caregivers: a protocol of a mixed method study in Sri Lanka and Poland
Source: Front Psychol. 2023 Dec 7;14:1194991. doi: 10.3389/fpsyg.2023.1194991 (PMC10740214; doi:10.3389/fpsyg.2023.1194991)
Supplement: Supplementary file 2 [file Presentation_2.PDF]

නිදන්ගත වකුගඩු රෝගයෙන් පෙළෙන රෝගීන්ගේ සහ ඔවුන්ගේ පවුල්වල මනෝ සමාජීය යහපැවැත්ම: සංසන්දනාත්මක අධ්‍යයනයක්

රෝගීන් සඳහා වූ සම්මුඛ සාකච්චා මග පෙන්වීම

සාමාන්‍යය තොරතුරු:

1. සහභාගිවන්නාගේ අංකය:
2. ස්ත්‍රී පුරුෂ භාවය:
3. වයස:
4. ජනවාර්ගික තත්ත්වය:
5. ආගම:
6. විවාහක තත්ත්වය:

විවාහකයි	වැන්දඹු	වෙන්ව ජීවත් වේ	දික්කසාද වී ඇත	අවිවාහකයි	අවිධිමත් සම්බන්ධතාවයක්

7. අධ්‍යාපනය:

ප්‍රාථමික	කනිෂ්ඨ ද්විතීක	ජේෂ්ඨ ද්විතීක	විශ්වවිද්‍යාල සහ තෘතීයික (උපාධිය, ශාස්ත්‍රපති, ආචාර්ය උපාධිය, වෙනත්)

8. පවුලේ සාමාජිකයින් කී දෙනෙකුට නිදන්ගත වකුගඩු රෝගය විනිශ්චය කර තිබේද?
9. ඔබගේ නිදන්ගත වකුගඩු රෝගයේ මට්ටම:
10. ඔබ කොපමණ කාලයක් නිදන්ගත වකුගඩු රෝගයෙන් පීඩා විඳිනවාද (කාල සීමාව)?
11. නිදන්ගත වකුගඩු රෝග විනිශ්චය කිරීමෙන් පසු ඔබට හැගෙන්නේ කුමක්ද?
12. ඔබ නිදන්ගත වකුගඩු රෝගී තත්වය තේරුම් ගන්නේ කෙසේද?
13. ඔබගේ මෙම රෝගී තත්වය එදිනෙදා ජීවිතයට (ආහාර, නින්ද, වැඩ, හැසිරීම් ආදියට) බලපා ඇත්තේ කෙසේද?
14. නිදන්ගත වකුගඩු රෝගය විනිශ්චය කිරීමෙන් පසු ඔබගේ පවුල සමඟ ඇති සබඳතාවලට සිදුවී ඇත්තේ කුමක්ද?

15. නිදන්ගත වකුගඩු රෝගය පිළිබඳ ඔබේ පවුලේ සාමාජිකයන් දක්වන ප්‍රතිචාරය ඔබ විස්තර කරන්නේ කෙසේද?
16. ඔබගේ රෝගී තත්ත්වය ඔබේ පවුලට බලපාන්නේ කෙසේද? (ආර්ථිකය, සෞඛ්‍යය, අධ්‍යාපනය සහ සබඳතා ආදිය)
17. නිදන්ගත වකුගඩු රෝගයෙ හි දුෂ්කරතා මගහරවා ගැනීම සඳහා ඔබේ ඥාතීන්, මිතුරන් සහ ප්‍රජාව විසින් ඔබට සහාය වන්නේ කෙසේද?
18. නිදන්ගත වකුගඩු රෝගී තත්ත්වය සමඟ කටයුතු කිරීමේදී ඔබ භාවිතා කරන පුද්ගලික නිපුණතා මොනවාද?
19. ඔබේ රෝගී තත්ත්වයට ප්‍රතිකාර කරන වෛද්‍ය ප්‍රජාව ඔබ විස්තර කරන්නේ කෙසේද?
20. නිදන්ගත වකුගඩු රෝගියෙකු ලෙස ඔබ සහභාගී වන්නේ කුමන ආකාරයේ සමාජ ක්‍රියාකාරකම් වලටද?
21. නිදන්ගත වකුගඩු රෝගය සමඟ කටයුතු කිරීමට උපකාර වන සම්පත් (පුද්ගලික, සමාජීය, සංස්කෘතික, ඖෂධීය, ආර්ථික) මොනවාද?
22. නිදන්ගත වකුගඩු රෝග තත්ත්වය සමඟ කටයුතු කිරීමට ආගමික සහ සංස්කෘතික චාරිත්‍ර ඔබට උපකාර වන්නේ කෙසේද?
23. නිදන්ගත වකුගඩු රෝගී තත්ත්වය සමඟ කටයුතු කිරීමට රජය ඔබට උපකාර කරන්නේ කෙසේද?
24. නිදන්ගත වකුගඩු රෝගී තත්ත්වය සමඟ කටයුතු කිරීමේදී ඔබට ඇති අභියෝග මොනවාද?
25. නිදන්ගත වකුගඩු රෝගීන්ට වඩා හොඳින් උපකාර කිරීම සඳහා වැඩිදියුණු කළ යුතු දේවල් මොනවාද?

නිදන්ගත වකුගඩු රෝගයෙන් පෙළෙන රෝගීන්ගේ සහ ඔවුන්ගේ පවුල්වල මනෝ සමාජීය යහපැවැත්ම: සංසන්දනාත්මක අධ්‍යයනයක්

<u>පවුල් පාදක සිද්ධි අධ්‍යයනය සඳහා සම්මුඛ සාකච්ඡා මග පෙන්වීම</u>

සාමාන්‍යය තොරතුරු:

1. සහභාගිවන්නාගේ අංකය:
2. ස්ත්‍රී පුරුෂ භාවය:
3. වයස:
4. ජනවාර්ගික තත්ත්වය:
5. ආගම:
6. විවාහක තත්ත්වය: (කරුණාකර පිළිතුර සලකුණු කරන්න)

විවාහකයි	වැන්දඹු	වෙන්ව ජීවත් වේ	දික්කසාද වී ඇත	අවිවාහකයි	අවිධිමත් සම්බන්ධතාවයක්

7. අධ්‍යාපනය:

ප්‍රාථමික	කනිෂ්ඨ ද්විතීක	ජ්‍යේෂ්ඨ ද්විතීක	විශ්වවිද්‍යාල සහ තෘතීයික (උපාධිය, ශාස්ත්‍රපති, ආචාර්ය උපාධිය, වෙනත්)

8. රෝගියාට ඇති සම්බන්ධය:
9. පවුලේ සාමාජිකයින් කී දෙනෙකුට නිදන්ගත වකුගඩු රෝග විනිශ්චය කර තිබේද?
10. ඔබගේ රෝගියාගේ නිදන්ගත වකුගඩු රෝගී මට්ටම:
11. පවුලේ සාමාජිකයා කොපමණ කාලයක් නිදන්ගත වකුගඩු රෝගයෙන් පීඩා විඳිනවාද (කාල සීමාව)?
12. ඔබේ පවුලේ සාමාජිකයා නිදන්ගත වකුගඩු රෝගියෙකු ලෙස හඳුනා ගැනීමෙන් පසු ඔබට හැගෙන්නේ කුමක්ද?
13. ඔබ නිදන්ගත වකුගඩු රෝගී තත්වය තේරුම් ගන්නේ කෙසේද?
14. ඔබගේ පවුලේ සාමාජිකයාගේ තත්වය ඔබගේ එදිනෙදා ජීවිතය හැඩගස්වා ඇත්තේ කෙසේද (ආහාර, නින්ද, වැඩ, හැසිරීම් ආදිය)?
15. ඔබගේ පවුලේ සාමාජිකයාට නිදන්ගත වකුගඩු රෝග විනිශ්චය කිරීමෙන් පසු රෝගියා සමඟ

ඔබේ සම්බන්ධතාවයට සිදුවී ඇත්තේ කුමක්ද?

16. නිදන්ගත වකුගඩු රෝගය පිළිබද ඔබේ පවුලේ සාමාජිකයන් දක්වන ප්‍රතිචාරය ඔබ විස්තර කරන්නේ කෙසේද?
17. ඔබේ රෝගියාගේ තත්ත්වය ඔබගේ පවුලට බලපා ඇත්තේ කෙසේද (ආර්ථිකය, සෞඛ්‍යය, අධ්‍යාපනය සහ සබඳතා ආදිය)?
18. නිදන්ගත වකුගඩු රෝගයෙ හි දුෂ්කරතා මගහරවා ගැනීම සඳහා ඔබේ ඥාතීන්, මිතුරන් සහ ප්‍රජාව විසින් ඔබට සහාය වන්නේ කෙසේද?
19. ඔබගේ රෝගියා සමඟ කටයුතු කිරීමේදී ඔබ භාවිතා කරන පුද්ගලික නිපුණතා මොනවාද?
20. ඔබේ රෝගියාට ප්‍රතිකාර කරන වෛද්‍ය ප්‍රජාව ඔබ විස්තර කරන්නේ කෙසේද?
21. නිදන්ගත වකුගඩු රෝගියාගේ උපස්ථායකයෙකු ලෙස, ඔබ කුමන ආකාරයේ සමාජ ක්‍රියාකාරකම්වලට සහභාගී වන්නේද?
22. නිදන්ගත වකුගඩු රෝගියා සමඟ කටයුතු කිරීමට ඔබට උපකාර වන සම්පත් (පුද්ගලික, සමාජීය, සංස්කෘතික, ඖෂධීය, ආර්ථික) මොනවාද?
23. නිදන්ගත වකුගඩු රෝගියාගේ තත්ත්වය සමඟ කටයුතු කිරීමට ආගමික සහ සංස්කෘතික චාරිත්‍ර ඔබට උපකාර වන්නේ කෙසේද ?
24. නිදන්ගත වකුගඩු රෝගියා සමඟ කටයුතු කිරීමට රජය ඔබට උපකාර කරන්නේ කෙසේද?
25. නිදන්ගත වකුගඩු රෝගියා සමඟ කටයුතු කිරීමේදී ඔබට ඇති අභියෝග මොනවාද?
26. නිදන්ගත වකුගඩු රෝගීන්ට වඩා හොඳ උපකාර කිරීම සඳහා වැඩිදියුණු කළ යුතු අවශ්‍යතා ගැන ඔබ සිතන්නේ කුමක්ද?
